# Electrochemical disinfection of toilet wastewater using wastewater electrolysis cell

**DOI:** 10.1016/j.watres.2016.01.040

**Published:** 2016-04-01

**Authors:** Xiao Huang, Yan Qu, Clément A. Cid, Cody Finke, Michael R. Hoffmann, Keahying Lim, Sunny C. Jiang

**Affiliations:** a Civil and Environmental Engineering, University of California, Irvine, CA 92697, United States; b Linde+Robinson Laboratories, California Institute of Technology, Pasadena, CA 91125, United States

**Keywords:** Electrochemical disinfection, Toilet wastewater, Wastewater electrolysis cell, Microbial pathogens, Disinfection byproducts, Solar energy

## Abstract

The paucity of proper sanitation facilities has contributed to the spread of waterborne diseases in many developing countries. The primary goal of this study was to demonstrate the feasibility of using a wastewater electrolysis cell (WEC) for toilet wastewater disinfection. The treated wastewater was designed to reuse for toilet flushing and agricultural irrigation. Laboratory-scale electrochemical (EC) disinfection experiments were performed to investigate the disinfection efficiency of the WEC with four seeded microorganisms (*Escherichia coli*, *Enterococcus*, recombinant adenovirus serotype 5, and bacteriophage MS2). In addition, the formation of organic disinfection byproducts (DBPs) trihalomethanes (THMs) and haloacetic acids (HAA_5_) at the end of the EC treatment was also investigated. The results showed that at an applied cell voltage of +4 V, the WEC achieved 5-log_10_ reductions of all four seeded microorganisms in real toilet wastewater within 60 min. In contrast, chemical chlorination (CC) disinfection using hypochlorite [NaClO] was only effective for the inactivation of bacteria. Due to the rapid formation of chloramines, less than 0.5-log_10_ reduction of MS2 was observed in toilet wastewater even at the highest [NaClO] dosage (36 mg/L, as Cl_2_) over a 1 h reaction. Experiments using laboratory model waters showed that free reactive chlorine generated *in situ* during EC disinfection process was the main disinfectant responsible for the inactivation of microorganisms. However, the production of hydroxyl radicals [•OH], and other reactive oxygen species by the active bismuth-doped TiO_2_ anode were negligible under the same electrolytic conditions. The formation of THMs and HAA_5_ were found to increase with higher applied cell voltage. Based on the energy consumption estimates, the WEC system can be operated using solar energy stored in a DC battery as the sole power source.

## Highlights

• A mobile toilet was developed for toilet wastewater treatment.
• 5-log_10_ reductions of bacteria and viruses were achieved within one hour.
• Reactive chlorine species were the main oxidants responsible for disinfection.
• The formation of organic disinfection byproducts was investigated.
• The treated water is safe for toilet flushing and irrigation.

## Graphical abstract

**Figure:**
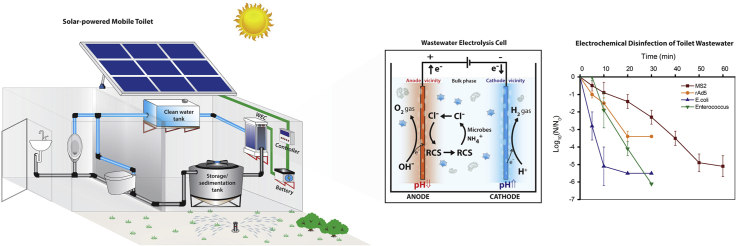

## 1 Introduction

In highly developed countries, flush toilets and well-managed sanitation systems have been used for more than 80 years. Wastewater is collected in massive sewer systems and subsequently treated at large-scale centralized wastewater treatment plants before discharged into receiving waters or land. Advanced, tertiary wastewater treatment plants are now capable of producing high quality reclaimed water for indirect or even direct potable reuse (Wetterau et al., 2013). In contrast, approximately 2.4 billion people in the developing world still lack the access to adequate sanitation facilities (WHO, 2015). The discharge of untreated or partially treated domestic wastewater to the aquatic environment severely threatens public health and socio-economic development. It is abundantly clear that, in many parts of the world, the infrastructure required for conventional centralized wastewater treatment systems is prohibitively expensive. The development of cost-effective, decentralized wastewater treatment systems is an important step toward the eradication of waterborne diseases and to ensure water sustainability in the developing world (Massoud et al., 2009).

Conventional flush toilet wastewater (i.e., a mixture of urine, feces and flushing water) is characterized by high levels of microbial contaminants (e.g., pathogenic viruses, bacteria and protozoa) derived from human excrement. Untreated toilet wastewater is considered as health hazard and potential vector of infectious waterborne diseases. On the other hand, toilet wastewater also contains high concentrations of macro- and micro-nutrients essential for plant growth. A large portion of these nutrients (e.g., nitrogen and phosphate) are contained in the liquid phase (Karak and Bhattacharyya, 2011). With proper treatment, it can be used as safe and cheap liquid fertilizer, which helps reduce the use of synthetic chemical fertilizer in developing countries (Drechsel et al., 2009). Therefore, the primary challenge of toilet wastewater reuse lies in how to effectively remove or inactivate microbial contaminants. Many disinfection technologies (e.g., chlorination, UV and ozonation) have been utilized in large-scale wastewater treatment plants. However, the adaptation of conventional disinfection systems to smaller decentralized plants is often difficult due to the financial constraints or technological barriers (Schmalz et al., 2009). In recent years, electrochemical (EC) disinfection has been considered as a viable alternative for decentralized wastewater treatment. EC disinfection has been reported to be capable of disinfecting a wide spectrum of microbial pathogens in various water matrices (Cano et al., 2012, Li et al., 2002). The on-site generation of disinfectants can be environmentally-sound and user friendly in terms of energy consumption and ease of operation. In addition, EC disinfection systems have the potential to be powered by solar energy, which is critical in many regions of the world where reliable energy supplies are not available (Cho et al., 2014b).

During EC disinfection, microorganisms are killed by a variety of oxidants that are produced during water electrolysis. When chloride is naturally present (e.g., seawater, toilet wastewater) or artificially added, reactive chorine species (RCS) such as free chlorine ([Cl_2_], [HOCl], [ClO^−^]) and chlorine radical species ([•Cl], [${\cdot {\text{Cl}}_{2}}^{-}$]) are generated and recognized as primary disinfectants. Human urine could serve as an excellent electrolyte as it contains high concentration of chloride (50–150 mM) (Kim et al., 2013, Putnam, 1971). In addition, reactive oxygen species (ROS) including hydroxyl radicals ([•OH]), hydrogen peroxide ([H_2_O_2_]), ozone ([O_3_]), and superoxide anion radicals ([•O_2_^−^]) generated during electrochemical water splitting can enhance the overall disinfection efficiency (Bergmann et al., 2008, Jeong et al., 2006). Another major factor governing the generation of oxidants is the composition of the anode (Jeong et al., 2009). Anodes made from antimony-doped tin oxide (ATO), PbO_2_ and boron-doped diamond (BDD) electrodes, are known to favor the formation of free [•OH]. In contrast, Pt-, IrO_2_-, RuO_2_- anodes, known as dimensionally stable anodes, form surface bound [•OH], which mediates the facile formation of RCS (Chen, 2004). Operational parameters, such as cell voltage, current density, reaction time, temperature, and pH are also important in the optimization of EC disinfection systems. In many previous studies, bacteria (usually *Escherichia coli* or total fecal coliform) were exclusively used as model microorganisms to evaluate the efficiency of EC disinfection systems since these microorganisms are specified in water quality standards or guidelines (Jeong et al., 2009, Schmalz et al., 2009). However, it is often unknown whether or not human enteric viruses are simultaneously inactivated along with the indicator bacteria during EC disinfection. Given the low infectious doses and potentially high resistance to commonly used disinfectants, viruses should be considered in the evaluation of disinfection efficacy of EC systems to ensure the quality of the treated water.

Furthermore, the formation of disinfection by-products (DBPs) in chlorination process is of concern because of their possible association with cancer and adverse reproductive outcomes (Nieuwenhuijsen et al., 2000). Depending on the reaction conditions, inorganic or organic DBPs may also be produced during EC disinfection. For instance, chlorite $\left(\left[{{\text{ClO}}_{2}}^{-}\right]\right)$ is known to be produced electrochemically either by oxidation of chloride or free chlorine ([HOCl] or [ClO^−^]) (Ghernaout et al., 2011). High overpotentials are often employed to electrochemically treat refractory organic pollutants. However, the use of high applied potentials in EC disinfection may lead to the formation of chlorate $\left(\left[{{\text{ClO}}_{3}}^{-}\right]\right)$, perchlorate $\left(\left[{{\text{ClO}}_{4}}^{-}\right]\right)$ persulfate $\left(\left[{{\text{SO}}_{5}}^{2-}\right]\right)$, and perphosphate $\left(\left[{{\text{PO}}_{5}}^{3-}\right]\right)$. These particular oxidants are toxic to humans and plants even at very low levels (Bergmann and Rollin, 2007, Kraft, 2008), and thus may restrict the reuse of the treated water. Even though the formation of chlorinated organics, such as trihalomethanes (THMs) and haloacetic acids (HAA_5_) during chlorination process is well known, the formation of organic DBPs during EC disinfection has not been studied extensively. Higher concentrations of chloride were found to produce higher concentrations of THMs during the EC disinfection of secondary wastewater effluent (Cano et al., 2012, Perez et al., 2010). Schmalz et al. (2009) reported levels as high as 1000 mg/L of organically-bound halogens were produced during EC disinfection as a direct function of the applied electric charge per volume (Q/V). Compared to domestic wastewater effluents, toilet wastewater may have higher concentration of dissolved organic carbon (DOC), which serves as precursors of organic DBPs. Consequently, higher concentrations of organic DBPs could be expected.

**Fig. 1:**
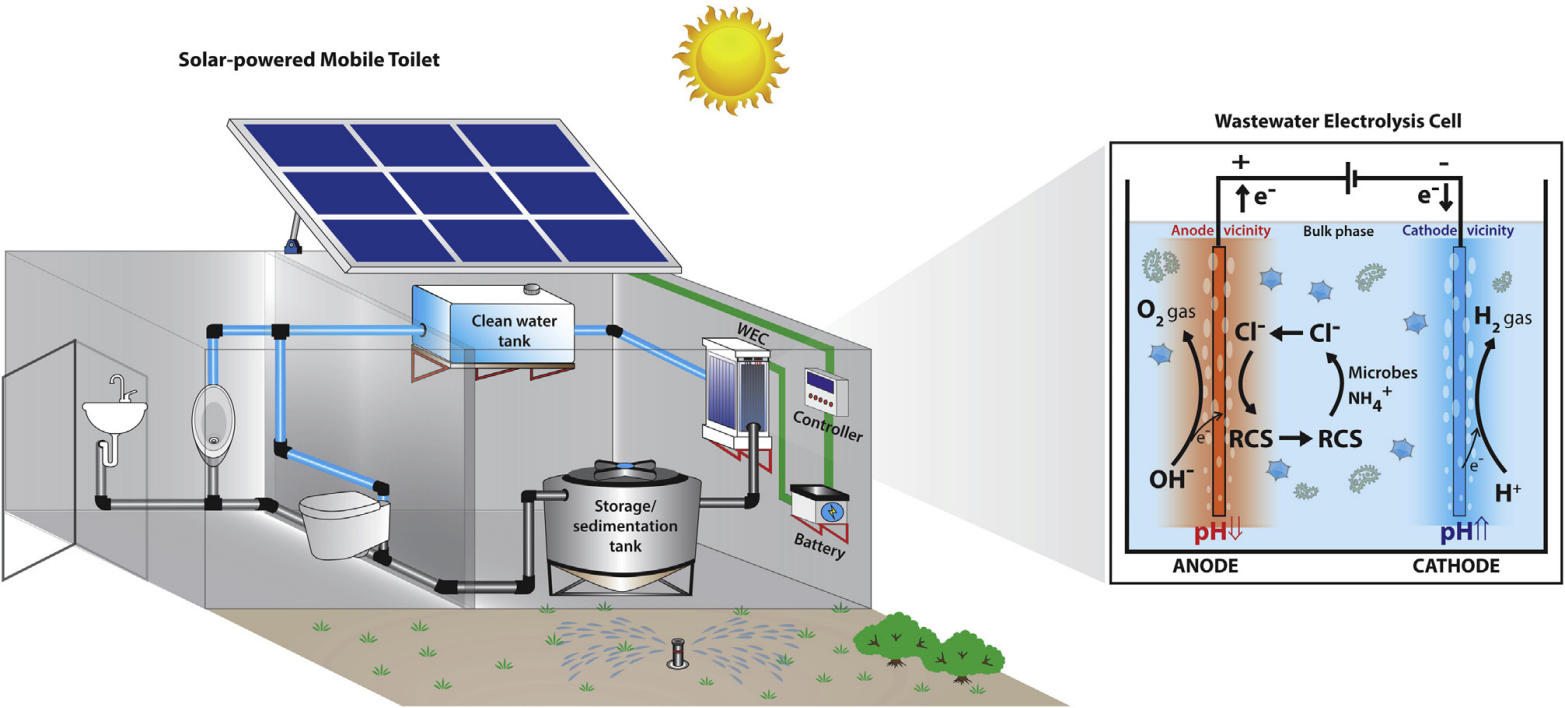
Schematic of a solar-powered mobile toilet using wastewater electrolysis cells (WEC) for toilet wastewater treatment.

Utilizing an array of mixed-metal oxide semiconductor anodes with stainless steel cathodes, we developed a variety of wastewater electrolysis cells (WEC), which can be powered by photovoltaic (PV)-panels for decentralized toilet wastewater treatment (Fig. 1). The treated wastewater was designed to reuse for toilet flushing and agricultural irrigation. The principle objective of this study is to assess and optimize the disinfection efficiency of the aforementioned WEC for toilet wastewater disinfection. In addition, the primary disinfection pathways for microbial inactivation were investigated along with the formation of THMs and HAA_5_ under typical operational conditions.

## 2 Materials and methods

### 2.1 Toilet wastewater and model waters

Fig. 1 shows the design of the pilot scale PV-powered self-contained mobile toilet system located at California Institute of Technology (Caltech). The EC reactor works inline, treating 20 L of wastewater each time in a batch model. The reactor is fed by the supernatant of the wastewater storage/sedimentation tank, which may also serve as a simple anaerobic digester. The treated effluent returns to the clean water tank for reuse. 40 L of toilet wastewater were collected from the storage/sedimentation tank for all the bench scale experiments. The pilot toilet system was in continuous operation when the wastewater sample was collected. The composition of the raw toilet wastewater is given in Table S1. All the microbial parameters were tested within 4 h of sample collection. Aliquots of wastewater were stored at 4 °C refrigerator before use. In addition to raw toilet wastewater, three laboratory model waters (MW1, 2, 3) were prepared and tested in order to characterize the role of RCS and ROS in EC disinfection. MW1 was phosphate buffered saline (PBS) solution containing only an inert electrolyte monopotassium phosphate ([KH_2_PO_4_]) in order to minimize the formation of oxidants other than ROS during EC reaction. MW2 was PBS buffer amended with 15 mM sodium chloride ([NaCl]). Free chlorine would equal to total chlorine in MW2 because of the absent of [NH_4_^+^]. In MW3, 15 mM of ammonium chloride ([NH_4_Cl]) was added to PBS buffer to provide [NH_4_^+^] and [Cl^−^], which ensured that free ammonia was readily available for reaction with [HOCl] during the first 30 min of EC reaction. All model waters were free of organics and other reducing agents. The general characteristics of the toilet wastewater and model waters were summarized in Table 1.

### 2.2 Model microorganisms

Microorganisms are naturally present in toilet wastewater. However, as shown in Table S1, the concentrations of culturable bacteria in the raw toilet wastewater collected from the pilot mobile toilet at Caltech were relatively low compared with those found in domestic wastewater. The low concentrations of microorganisms were likely due to the low usage of the toilet for defecation, the reuse of EC treated water for toilet flush (which contains chlorine residual) and the long water retention time (>20 days) in the storage/sedimentation tank (anaerobic digestion). The concentrations of indigenous microorganisms become even lower after 4 °C refrigerator storage (data not shown). To maintain consistent initial levels of target microorganisms and keep other physiochemical parameters (e.g., pH, chloride, and ammonia) comparable, we chose to seed pure-cultured model bacteria or viruses to aliquots of the single batch of 40 L wastewater sample before each trail. *E. coli K12* (ATCC 10798) and *Enterococcus faecalis* (ATCC 29212) were employed as model bacteria in the disinfection experiments. Both bacteria were cultured in Luria–Bertani broth at 37 °C for 18 ± 2 h. The bacterial suspensions were centrifuged at 3000 × *g* for 15 min and re-suspended in 1 × PBS, which were used as bacterial stock. Before each experiment, a freshly prepared 0.1–1 mL bacterial stock suspension was seeded to water samples to give an approximate final concentration of 10^5^–10^6^ CFU/mL. *E. coli* and *Enterococcus* concentrations in the water samples were quantified using membrane filtration via US EPA method 1063 and method 1600, respectively. Part of the samples were also analyzed using a flow cytometer (FCM, Accuri Cytometers, Ann Arbor, MI, USA) to assess the bacterial nucleic acid injuries induced by EC disinfection. The indigenous bacterial cells in the toilet wastewater samples were negligible in FCM analysis, because the concentrations of seeded bacteria were more than 100 times higher than those remaining in the 4 °C stored toilet wastewater sample. The samples were fixed with 2% glutaraldehyde (final concentration) and stained with 0.5x SYBR-Gold (Invitrogen Corp., Carlsbad, CA). The FCM instrument setting and data acquisition procedure were described in detail in our previous study except the threshold was set at 10,000 in fluorescence channel 1 (FL1) (Huang et al., 2015). Green fluorescence and side scatter (SSC) light were collected in the FL1 channel (533 ± 30 nm) and the SSC channel on a logarithmic scale, respectively. Data analysis was carried out using the BD CFlow® software. FL1 vs. SSC density plots were used to differentiate different bacterial populations as well as the background noise.

Coliphage MS2 (ATCC 15597-B1) was propagated using *E. coli* – 3000 (ATCC 15597) as host. Briefly, 0.1 mL (10^7^ pfu/mL) MS2 was inoculated into 20 mL actively growing *E. coli* host suspension. The infected bacteria were continuously aerated at 37 °C for 36 h. The host-associated MS2 suspension was then centrifuged at 3000 × *g* for 20 min to remove the bacterial cells and debris. The supernatant containing the MS2 phages was further purified by 0.2 μm syringe filter (GE Whatman, Pittsburgh, PA). The filtrate was diluted 1,000x with 1 × PBS and stored in −80 °C freezer, which was used as MS2 stock. The seeding level of MS2 was 10^5^ to 10^6^ PFU/mL. The concentration of MS2 in water samples was titrated by the double agar layer method (Clokie, 2009).

Recombinant adenovirus serotype 5 (rAd5) with the E1A gene replaced by the green fluorescent protein (GFP) gene was also employed as model virus in the seeding study. A human embryonic kidney cell line (HEK-293A) was used as rAd5 host for the propagation and the quantification of rAd5. Viral infectious units were quantified using a flow cytometer by detecting the GFP positive cells as described in detail by Li et al. (2010). The seeding level of rAd5 was 10^4^ PFU/mL due to the relatively low concentration of rAd5 stock.

### 2.3 Experimental procedures

EC disinfection experiments were carried out in a bench scale WEC with a working volume of 250 mL. The electrode module used for this study consists of a semiconductor anode (Nanopac, Korea, 13.5 × 6.7 cm) and a stainless steel cathode (Hastelloy C-22, 13.5 × 6.7 cm). The details about the preparation and characteristics of the BiOx/TiO_2_ anode can be found in our previous reports (Cho et al., 2014a, Cho et al., 2014b). The electrode pairs were installed in the reactor with a separation between anode and cathode of 0.5 cm. The setup was connected to a direct current power supply (HP-6236B Triple Output Power Supply, Palo Alto, CA) and operated in potentiostatic mode at 3 V, 4 V and 5.5 V providing current density values of 0.39, 1.2 and 2.4 mA/cm^2^. All the experiments were carried out at room temperature (21 ± 0.5 °C). Before water electrolysis, target concentration of model microorganisms were seeded into 250 mL toilet wastewater or laboratory MWs and stabilized for 30 min. In a sub-set of the experimental trails using MWs, an excessive amount of *tert*-Butyl alcohol (*t*-BuOH) (30 mM), a well-known [•OH] scavenger (*k*·[•OH]+*t*-BuOH = 5 × 10^8^ M^−1^ s^−1^), was added to raw water to assess the presence and role of [•OH] in EC disinfection (Enami et al., 2014, Jeong et al., 2006). During the EC reaction, the electrolyte was well mixed by a magnetic stir bar. Samples were withdrawn at time intervals to measure the concentrations of oxidants or to count the number of viable microorganisms. The change of the volume and wetted electrode surface area due to the sample withdraw from the reactor was negligible. For microorganism enumeration, samples were quenched immediately with excess [Na_2_S_2_O_3_] (10 mM) to eliminate the residual disinfectants (Jeong et al., 2009). The level of inactivation was expressed as a log reduction of the microbe survival ratio (N/N_0_) during the disinfection experiments. All experiments were repeated at least twice to ensure repeatability. In preliminary disinfection experiments with toilet wastewater, inactivation of microorganisms was not observed under 3 V, while the inactivation rate of MS2 was similar under 4 V and 5.5 V. Thus, in the later EC disinfection experiments, the applied cell voltage was set at 4 V unless specified.

Chemical chlorination (CC) was carried out for comparison in order to investigate the role of different chlorine species (free/combined chlorine) in EC disinfection. Similar to the EC disinfection experiments, microorganisms were seeded in water samples contained in sterile capped glass bottles. Samples were stabilized for 30 min before adding freshly prepared [NaClO] stock solution (1000 mg/L) to the desired final concentrations. A magnetic stir bar was used to keep the samples well mixed over the course of reactions. Water samples were taken and tested following the same procedures as in EC disinfection experiments to quantify the chlorine residual and viable microorganisms.

### 2.4 Chemicals and analysis

The concentration of total chlorine and free chlorine in water samples were determined as mg/L [Cl_2_] by N,N-diethyl-p-phenylenediamine (DPD) colorimetric method using a OMEGA photometer (HHWT-11). It is important to mention that in EC disinfection, besides chlorine, other potential oxidants (e.g., [O_3_], [H_2_O_2_], [ClO_2_]) produced by EC reaction can also oxidize DPD to form the semiquinoid cationic Würster dye that accounts for the magenta color in the colorimetric test, and thus the total chlorine results in fact reflect the total oxidizing capacity of EC produced oxidants in the sample (expressed as mg/L Cl_2_) (Danial, 2002, Schmalz et al., 2009).

The formation of THMs (CHCl_3_, CHBrCl_2_, and CHBr_2_Cl) and HAA_5_ (C_2_HCl_3_O_2_, C_2_H_2_Cl_2_O_2_, C_2_H_3_ClO_2_, C_2_H_2_Br_2_O_2_, and C_2_H_3_BrO_2_) was measured by an EPA certified laboratory using US EPA method 524.2 and US EPA method SM 6251 B by gas chromatography (Eurofins Eaton Analytical, Inc., Monrovia, CA). Briefly, unseeded toilet wastewater was treated with WEC under applied cell voltage of 4 V and 5.5 V for 60 min. At the end of the reaction, the treated water was divided into two portions. The first portion was immediately transferred to sampling bottles with quencher [Na_2_S_2_O_3_] (for THMs) or [NH_4_Cl] (for HAA_5_) to stop the reaction (0 h samples). The other portion was incubated in amber glass bottles capped with Teflon-faced septa at room temperature (21 ± 0.5 °C) in the dark for 24 h before transferred to sampling bottles (24 h samples). For comparison, the formation of DBPs during CC disinfection were also tested with [NaClO] addition at the total chlorine concentration of 5 mg/L (as Cl_2_), which was at equivalent to the chlorine concentration at the end of 60 min EC disinfection under 4 V. Similarly, 0 h and 24 h samples were collected and tested in the same way as in EC disinfection.

## 3 Results

### 3.1 Electrolysis production of oxidants in toilet wastewater and model waters

**Fig. 2:**
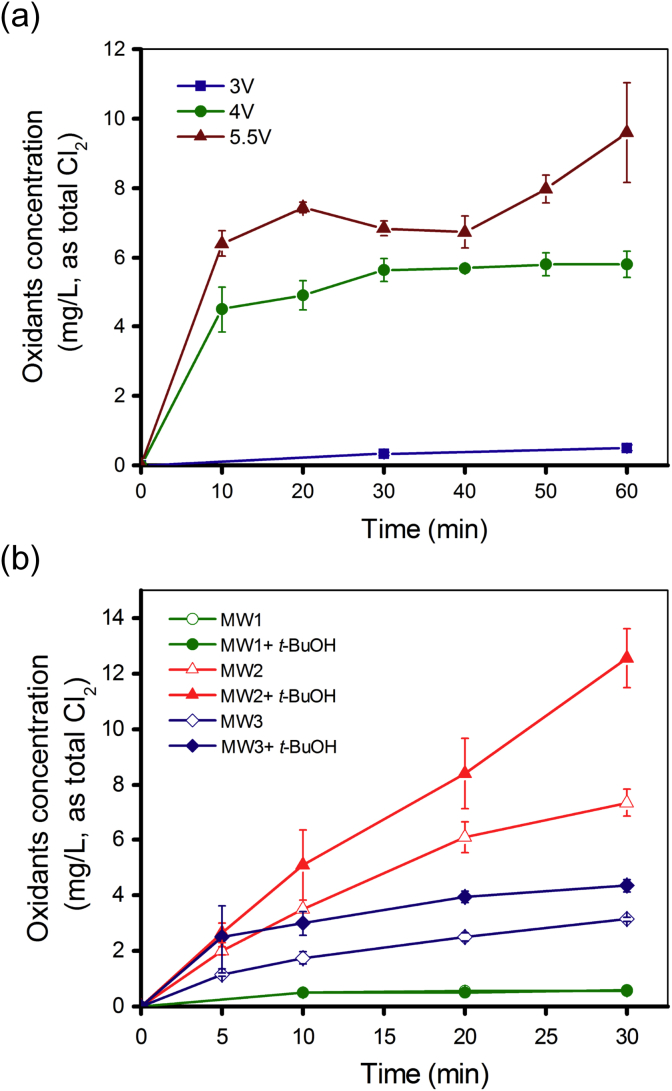
Oxidants generation during electrochemical (EC) reaction (expressed as mg/L, Cl_2_) in (a) toilet wastewater under different applied cell voltage and (b) model waters at applied cell voltage of 4 V (MW1: PBS; MW2: PBS+15 mM NaCl; MW3: PBS+15 mM NH_4_Cl).

The influence of applied cell voltage on oxidants generation in toilet wastewater is shown in Fig. 2a. Very limited oxidants (<0.5 mg/L) were detected under the applied cell voltage of 3 V. At cell voltage of 4 V, the concentration of oxidants increased to 4 mg/L within 10 min and stabled at 5–6 mg/L during rest of reaction time. When the applied cell voltage was raised to 5.5 V, the oxidants generation curve showed resemblance to the direct chlorination process (breakpoint chlorination). At both 4 V and 5.5 V, free chlorine (>1 mg/L) was detected by the DPD method after 5 min of reaction (data not shown). Due to the interference of chloramines, DPD method cannot precisely quantify the free chlorine level in the grab samples (the magenta color increased with time resulting in the overestimation of free chlorine) (Spon, 2008). However, the existence of free chlorine before breakpoint (with the present of free ammonia in the system) during the EC reaction was confirmed in our 20 L prototype WEC by an online free chlorine probe (Chemtrac, Norcross, GA). The kinetics of free chlorine evolution and pH changes in the prototype WEC are shown in Fig. S1.

The analysis of individual oxidants during electrolysis of toilet wastewater is extremely difficult due to the complexity of the water as well as the potentially fast reactions of oxidants with ammonia or other reducing substances (e.g., organic matter). The EC experiments using laboratory model waters provided additional evidence for explaining the role of different oxidants in disinfection (Fig. 2b). In the absence of [Cl^−^], less than 0.5 mg/L total oxidants were detected in MW1. In MW2 and MW3, total chlorine concentration steadily increased with the reaction time (Fig. 2b), although the chlorine evolution rate in MW3 was obviously slower than that in MW2. The addition of excess *t*-BuOH (30 mM) was intended to quench [•OH] in order to inhibit the chlorine production mediated by [•OH]. Unexpectedly, *t*-BuOH in fact promoted the chlorine evolution in both MW2 and MW3 (Fig. 2b). However, the similar stimulating effect was not observed in MW1, in which less than 0.5 mg/L total oxidants were detected.

### 3.2 Inactivation of microorganisms in toilet wastewater by EC and CC disinfection

**Fig. 3:**
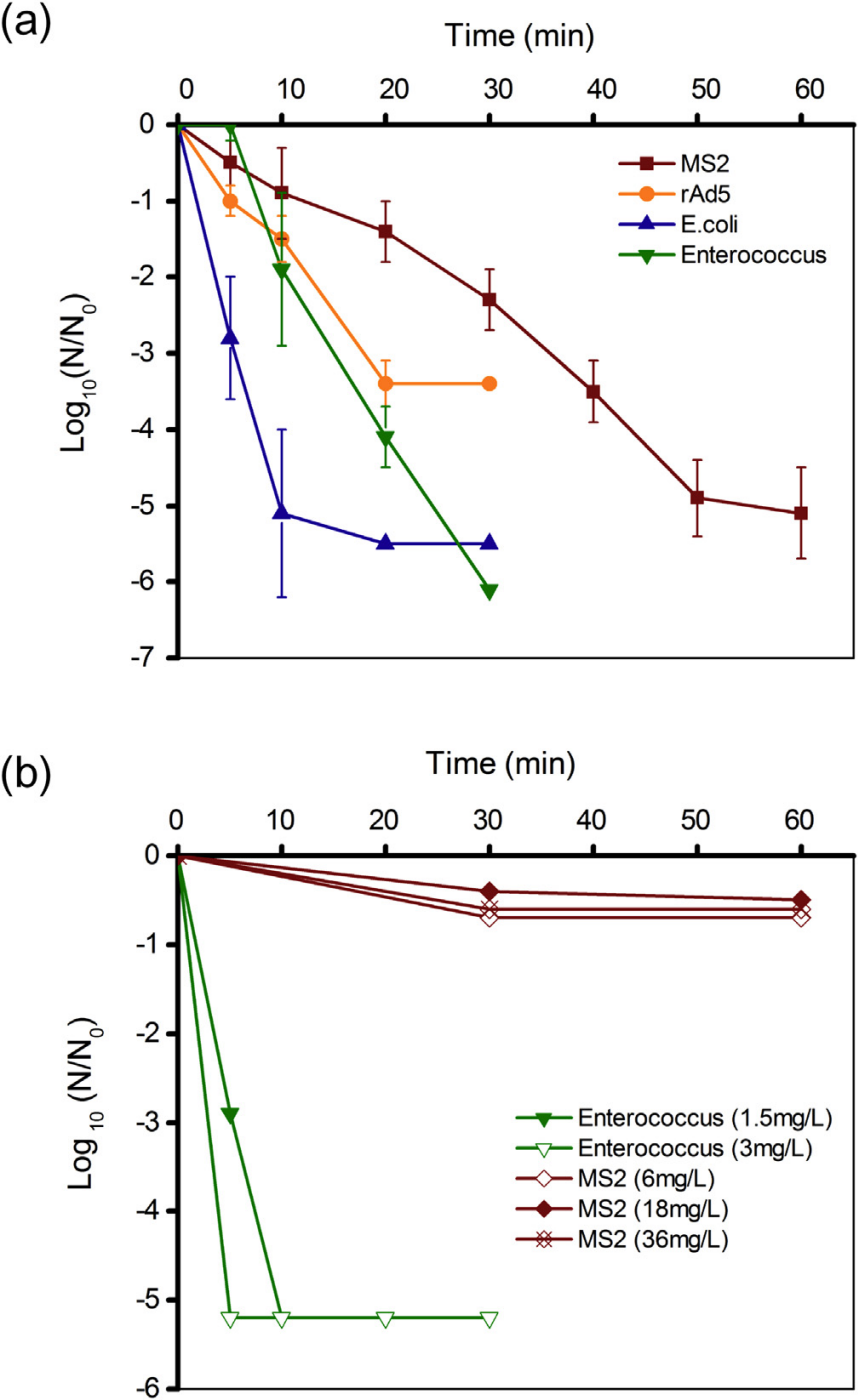
Inactivation kinetics of microorganisms in toilet wastewater by (a) electrochemical (EC) disinfection at applied cell voltage of 4 V and (b) chemical chlorination (CC) disinfection using different concentrations of [NaClO] (as mg/L Cl_2_, indicated in the legend).

The successful inactivation of all four seeded microorganisms was achieved within 60 min at the applied cell voltage of 4 V (Fig. 3a). *E. coli* were highly susceptible to EC disinfection. A 2-log_10_ reduction of *E. coli* was observed within the first 5 min and no viable *E. coli* was detected after 20 min of reaction. For *Enterococcus*, a clear lag phase (0–5 min) prior to the expected pseudo first-order kinetics was observed indicating their mild resistance to low level of EC produced oxidants. All *Enterococcus* were inactivated to below the detection limit after 30 min. The differences in resistance to EC disinfection between *E. coli* and *Enterococcus* were also reflected by the FCM results (Fig. S2). After 30 min of EC reaction, no significant change of the particle fluorescence intensity of *Enterococcus* cells was observed (R1, Fig. S2b), while about 40% of the *E. coli* cells shifted from high fluorescence region (R2, Fig. S2a) to lower fluorescence region (R3, Fig. S2a). For both types of bacteria, the fluorescence total cell counts decreased about 10–15% indicating they may have become ghost cells (lost DNA integrity) or been totally destroyed after EC disinfection. Due to the lower seeding level, only a 3-log_10_ reduction of rAd5 was shown in Fig. 3a, yet it demonstrated higher resistance than both types of bacteria. The inactivation of MS2 was the slowest taking about 60 min to reach a 5-log_10_ reduction.

In the comparative CC disinfection experiments, a 5-log_10_ reduction of *Enterococcus* was achieved within 10 min at the total chlorine dosing level of 1.5 mg/L (Fig. 3b). On the contrary, the inactivation rate of MS2 was significantly lower (<0.5-log_10_) even at the highest [HClO] dosage (36 mg/L, as Cl_2_). Considering the [NH_4_^+^] concentration in the toilet wastewater (Table 1), monochloramine was the main disinfectant under all [NaClO] dosing levels, although free chlorine may exist momentarily in the system at the beginning of [NaClO] dosing.

### 3.3 Inactivation of MS2 by EC and CC in model waters

**Fig. 4:**
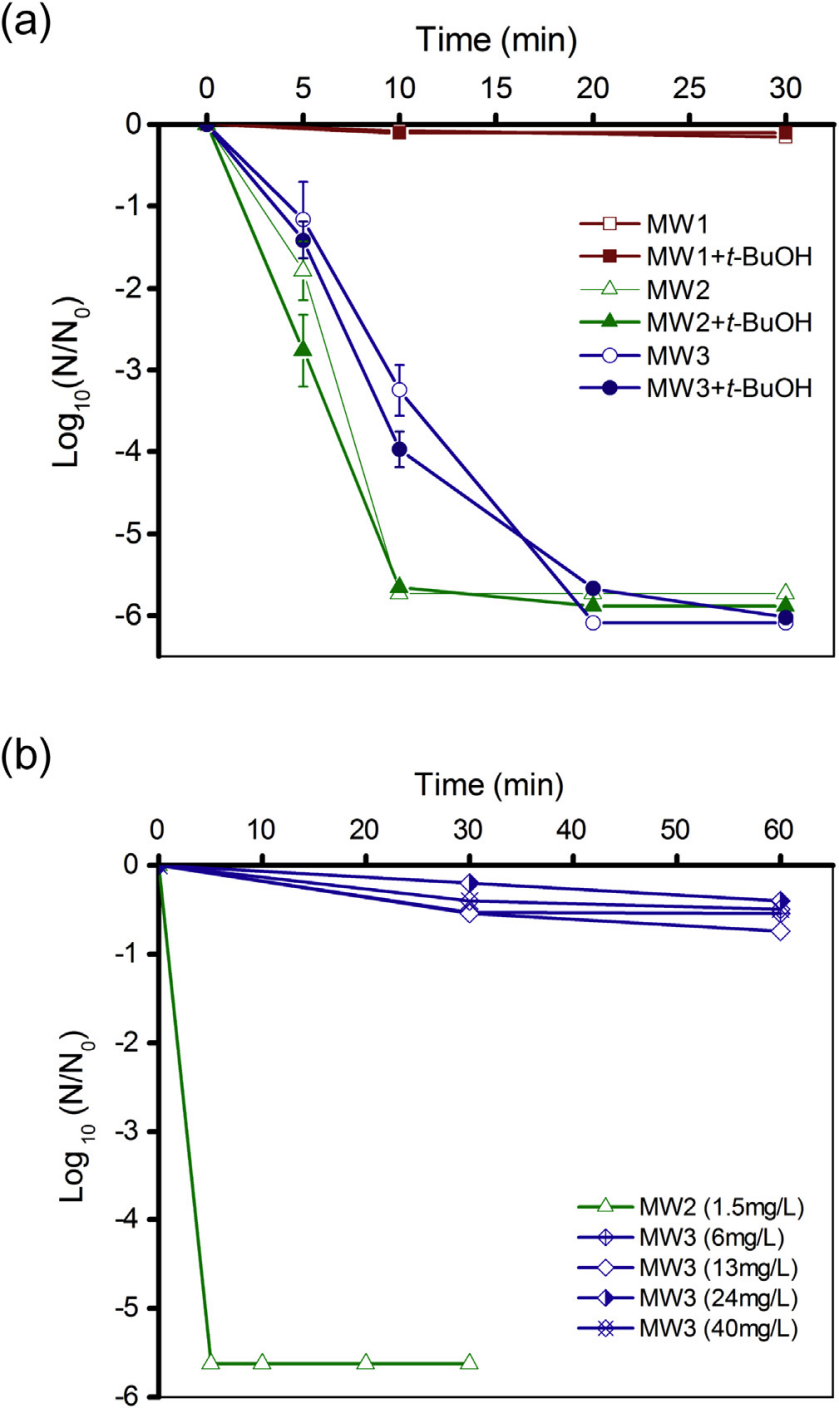
Inactivation kinetics of MS2 in model waters by (a) electrochemical (EC) disinfection (applied cell voltage: 4 V) and (b) chemical chlorination (CC) disinfection using different concentrations of NaClO (as mg/L Cl_2_, indicated in the legend).

The inactivation kinetics of MS2 by EC disinfection in MWs are shown in Fig. 4a. No MS2 reduction was observed in MW1 electrolysis, while a 5-log_10_ reduction of MS2 were achieved within 10 min and 20 min in MW2 and MW3, respectively. The addition of *t*-BuOH (30 mM) slightly enhanced the inactivation rate in MW2 and MW3, but it did not change the inactivation rate of MS2 in MW1. Fig. 4b shows the inactivation of MS2 in MWs by CC disinfection. Similar to the toilet wastewater (Fig. 3b), in MW3, less than 0.5-log_10_ reduction of MS2 was observed at the chlorine concentration as high as 40 mg/L (combined chlorine, as Cl_2_). In contrast, in the absence of [NH_4_^+^], a 5-log_10_ reduction of MS2 was achieved within 5 min in MW2 at the chlorine concentration of 1.5 mg/L (free chlorine, as Cl_2_) (Fig. 4b).

### 3.4 The formation of trihalomethanes (THMs) and haloacetic acids (HAA_5_)

**Fig. 5:**
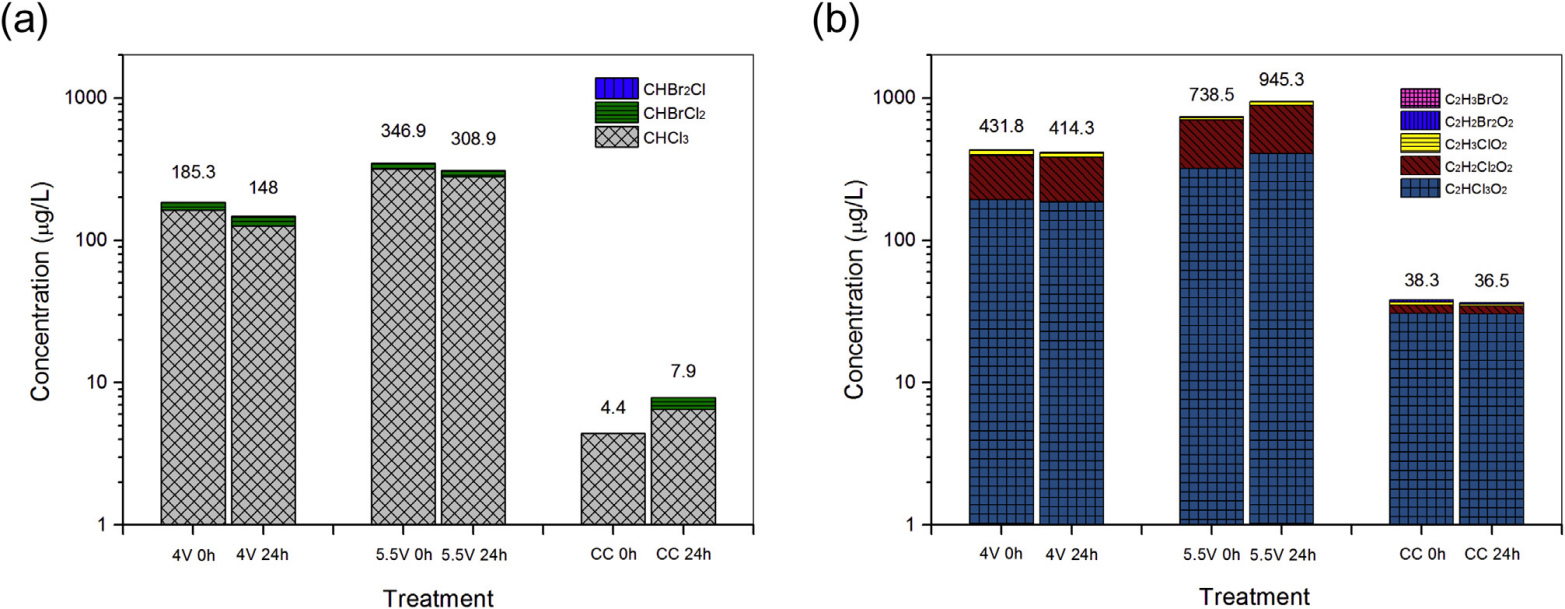
The formation of (a) THMs and (b) HAA_5_ in toilet wastewater after one hour electrochemical (EC) disinfection under applied cell voltage of 4 V and 5.5 V and one hour chemical chlorination (CC) disinfection using NaClO (5 mg/L as Cl_2_). 0 h samples were collected into sampling bottles with quenchers immediately at the end of reactions, while the 24 h samples were same samples collected after a 24 h incubation time.

Fig. 5 shows the formation of DBPs in toilet wastewater after EC and CC disinfection. It is found that under both treatment processes, the composition of THMs and HAA_5_ appears to be quite similar. For example, trichloromethane [CHCl_3_] accounted for more than 90% of THMs, while trichloroacetic acid [C_2_HCl_3_O_2_] and dichloroacetic acid [C_2_H_2_Cl_2_O_2_] were the most abundant compounds of HAA_5_. Only a small amount of brominated DPBs were detected in the treated water samples (less than 5%). However, the results also demonstrated that the formation of DPBs during EC disinfection was significantly higher than in CC disinfection, although the measured total chlorine concentrations in EC disinfection (4 V, 60 min) was equivalent to that in CC disinfection (5 mg/L, as Cl_2_). For EC disinfection, when the applied cell voltage was raised from 4 V to 5.5 V, the concentrations of THMs and HAA_5_ were almost doubled (Fig. 5). The impact of incubation time on DBPs formation was limited. Most of the DBPs had been generated during the reaction (0 h samples), except for the THMs formed during CC disinfection, which increased about 45% after a 24 h-incubation.

## 4 Discussion

### 4.1 Oxidant generation and energy requirements

For effective RCS production, the actual anodic potential needs to be higher than the chlorine evolution overpotential (*E*^*0*^ [Cl_2_]/[Cl^−^] = 1.36 V). Based on the current data, the applied cell voltage of 4 V was necessary to overcome the ohmic resistance to have chlorine generation in the toilet wastewater (Fig. 2a). The [Cl_2_] produced by EC reaction quickly reaches equilibrium with [HOCl] and [ClO^−^] in the bulk aqueous phase. At the same time, further oxidation of free chlorine ([HOCl] or [ClO^−^]) to $\left[{{\text{ClO}}_{3}}^{-}\right]$ and $\left[{{\text{ClO}}_{4}}^{-}\right]$ can happen considering the relatively lower reducing potential for the redox couples ($E^{0}\left[{{\text{ClO}}_{3}}^{-}\right]/\left[{\text{ClO}}^{-}\right]=0.94\;\text{V}$, $E^{0}\left[{{\text{ClO}}_{4}}^{-}\right]/{{\text{ClO}}_{3}}^{-}=1.23\;\text{V}$). Cho et al. (2014b) reported that, $\left[{{\text{ClO}}_{3}}^{-}\right]$ was the main inorganic chlorinated byproduct in the electrolysis of municipal wastewater. Trace-levels of [ClO_2_] may also be produced during the reaction, which is depending on the pH. Acidic solutions were reported to favor the formation of [ClO_2_], while alkaline conditions support the production of $\left[{{\text{ClO}}_{3}}^{-}\right]$ (Ghernaout et al., 2011). Although higher applied cell voltage can increase the [Cl_2_] generation rate and maintain the total oxidants at a higher level in the bulk solution (Fig. 2a), it may also increase the formation of $\left[{{\text{ClO}}_{3}}^{-}\right]$ and other highly oxidized oxidants. These oxidants are toxic to plants even at low concentration, which may limit the potential uses of the treated water. Therefore, higher applied voltage or extended reaction time should be avoided once the disinfection goal is achieved. The oxidants generated during the electrolysis of MW samples are consistent with the characteristics of active electrodes. [•OH] and other ROS were not efficiently produced by the BiOx/TiO_2_ anode as indicated by the low level of oxidants detected during the electrolysis of inert sample, MW1 (Fig. 2b). As a consequence, no inactivation of MS2 was observed in MW1 (Fig. 4a). The result indicates [Cl^−^] is an indispensable component for the current EC disinfection system. The increase of chlorine evolution rate in MW2 and MW3 with the addition of the [•OH] scavenger *t*-BuOH conflicts with a previous report, which showed no significant changes in chlorine evolution rates were observed with active anode (Jeong et al., 2009). The possible reason could be the reaction of *t*-BuOH with the surface bounded [•OH], which in return enhanced the oxidation of [Cl^−^] at the interface due to electron transfer.

Based on the current setting for microbial inactivation, the energy requirement of the WEC can be calculated using equation $\varepsilon =\frac{E_{cell}\int i\left(t\right)}{V}\;$, where *E*_*cell*_ is the applied cell voltage, *i* is the current and *V* is the volume of reactor. Under the optimal condition (*E*_*cell*_ = 4 V, *i* = 0.125 A), to achieve a 5-log_10_ reduction of the MS2 (conservative model microorganism), the reaction time was about 1 h and thus the energy consumption was estimated to be 2 Wh/L. Considering the ohmic losses and the power required for the circulation pumps and electronic controller, the actual energy consumption of the Caltech 20 L prototype WEC was estimated to be 13–15 Wh/L (260–300 Wh/reaction for 20 L). The energy requirement indicates that the WEC can use inexpensive commercial PV-panels as the sole power source (e.g., Sonali 300W, Miami Gardens, FL). As there is no need for external power supply and supporting chemicals, the system is suitable for decentralized toilet wastewater disinfection.

### 4.2 Disinfection mechanisms

The inactivation of bacteria during disinfection process can be generally explained by two types of damages to bacterial cells. First, disinfectants can react with cell surface components causing cell membrane permeability changes or the malfunction of enzymatic transport systems. Second, impairments to the intracellular components, especially the loss of DNA integrity, could be introduced with or without obvious cell surface damages (Cho et al., 2010). Certain disinfectants cause more significant damages to either the cell surface or internal components, but these two types of damages are not exclusive, which would also strongly depend on the Ct-value (disinfectant concentration × reaction time) as well as the type of bacterial cells. During EC disinfection, *E. coli* and *Enterococcus* behaved quite differently, especially in the beginning of the reaction when the concentration of oxidants was below 2 mg/L (0–5 min, 4 V). Similar results were found in the study on traditional chlorination disinfection process (Tree et al., 2003). The different inactivation kinetics between the two indicator bacteria are likely related to their cell surface structure differences (Gram-negative *vs.* Gram-positive bacteria), since at low chlorine concentration (<0.5 mg/L, as Cl_2_), damages of chlorine were reported mainly to the cell surfaces (Phe et al., 2005). When the chlorine concentration exceed the threshold (free chlorine, between 1.5 and 3 mg/L as Cl_2_), severe damages to bacterial genomes could happen (Phe et al., 2005, Ramseier et al., 2011). This effect was indicated by the bacterial clusters shift (from the high fluorescence region to the low fluorescence region) and particle count loss in the FCM analysis, because damaged bacterial genomic DNA cannot be effectively stained by fluorescent dyes. In the current study, although both *E. coli* and *Enterococcus* became non-culturable after 30 min of EC reaction, significant fluorescent intensity decrease was only observed for *E. coli* cells (Fig. S2, R2 to R3). No such changes were observed for *Enterococcus* cells (Fig. S2, R1). This result indicates that most of the *Enterococcus* cells still maintained their genomic DNA integrity. Therefore, a longer treatment time would be necessary to ensure all the non-culturable bacteria were actually dead as injured bacterial cells have been known to be able to repair certain damages when the environmental forcing factors are removed (Li et al., 2013). Also, the maintenance of a chlorine residual (e.g., 2 mg/L) in the clean water storage tank (Fig.1) is suggested, which will restrict the regrowth of bacteria after EC treatment.

In addition to bacteria, pathogenic viruses raise even greater concerns in toilet wastewater reuse. Current results confirmed that neither *E. coli* nor *Enterococcus* is an adequate indicator for virus inactivation during toilet wastewater disinfection. The fate of human adenovirus (a double stranded DNA virus) was for the first time studied in an EC disinfection system. The rAd5 demonstrated higher resistance compared to both bacterial indicators (Fig. 3a). Virus inactivation is more complicated than bacteria by the fact that highly related viruses can exhibit different disinfection kinetics when treated by the same disinfection procedure. For example, human adenovirus serotype 40 (HAdV40) and HAdV41 were reported to be more susceptible to monochloramine disinfection than HAdV2 (Cromeans et al., 2010). These variable responses suggest that even minor variations in structural or genomic components can have a remarkable impact on virus resistance to inactivation. Studies have also shown that seeded laboratory-cultured viruses are less resistant to disinfection processes compared to their indigenous counterpart, because indigenous strains are often embedded in biofilm or attached to suspended solids, which may shield the virus against disinfectants (Tree et al., 2003). All these factors highlighted the importance of choosing a conservative virus surrogate in evaluating the disinfection capability of EC systems. In this context, MS2 should be an ideal candidate as it demonstrated higher resistance than rAd5 in the current study and had been reported to be more resistant than poliovirus (Tree et al., 2003), coxsackieviurs (Tree et al., 2003), and hepatitis A virus (Casteel et al., 2008) to a variety of disinfectants. When a 5-log_10_ reduction of MS2 is achieved after EC disinfection, other bacteria and viruses in the toilet wastewater should also be effectively inactivated and the health risks associated with non-potable water reuse would be significantly reduced.

The comparative experiments between EC and CC disinfection clearly demonstrated the advantage of EC disinfection for virus disinfection in toilet wastewater. In [NH_4_^+^] free MW1, a 5-log_10_ reduction of MS2 was achieved at free chlorine level of 1.5 mg/L within 5 min. The result showed that MS2 was in fact very sensitive to free chlorine. Wigginton et al. (2012) studied the damages of free chlorine to MS2 and found it is a non-discriminative oxidant causing the losses of functions such as genome-mediated replication and protein-meditated injection. The current results indicate that the chlorine species (free or combined chlorine) were the decisive factor controlling the virus disinfection efficacy. Compared to free chlorine, monochloramine is a much weaker disinfectant, which is only effective for bacterial inactivation, but not effective for viruses (Fig. 3b). When free [Cl_2_] to [${{\text{NH}}_{4}}^{+}$] ratio (weight ratio Cl_2_:NH_3_–N) was less than 5, the conversion of free chlorine to monochloramine can be completed within seconds under optimal conditions (pH = 8.4; 25 °C) (Kirmeyer et al., 2004). This explained the limited virus reduction observed during CC disinfection in the toilet wastewater and MW3. For CC disinfection, free chlorine residual can be created by adding extra amount of [NaClO] to bring the system past the breakpoint (weight ratio Cl_2_:NH_3_–N>9:1). However, this may not be practical for raw toilet wastewater disinfection, as the total chlorine residual would be too high for any reuse applications (>500 mg/L, as Cl_2_). In contrast, free chlorine was always present during toilet wastewater electrolysis even with the present of free ammonia, because it was continuously produced on the surface of anode. Free chlorine may react with viruses before it is converted to chloramines. In addition, a local pH decrease at the anode surface occurs due to the production of [H^+^] through [O_2_] evolution. Lower pH can significantly reduce the chlorine-ammonia reaction rate. Acidic conditions also favor the formation of neutral [HClO], a more effective disinfectant than the negatively charged [ClO^−^]. Lastly, ammonia was converted to $\left[{{\text{NO}}_{3}}^{-}\right]$ and [N_2_] during EC reaction, and thus the [Cl_2_] to [NH_4_^+^] ratio was increasing with reaction time. Besides free chlorine, [ClO_2_] may also contribute to the MS2 inactivation in EC disinfection. [ClO_2_] have been shown to cause the degradation of viral proteins and thus inhibit the host-cell recognition and binding (Wigginton et al., 2012). However, considering the low concentration of [ClO_2_] during the reaction, its virucidal effect may be limited.

### 4.3 DBPs formation

Previous studies have shown that the formation and distribution of DBPs in chlorinated waters are dependent on water source (levels of organic precursors), contact time, pH, and the bromide concentration (Hua and Reckhow, 2008, Hua et al., 2006). In the present study, the DBPs formation observed after EC and CC disinfection can be mainly explained by the difference of chlorine species present in the systems. Although the measured total chlorine residuals were the same in both systems (EC 4 V *v.s.* CC), as discussed before, free chlorine was constantly present in the EC system during toilet wastewater electrolysis. It may react with organic matter to form DPBs before it was converted to chloramines. However, in CC disinfection, the high reaction rate between free chlorine and ammonia indicates that the competition reactions between free chlorine and organic matter were suppressed. This postulation is supported by previous studies showing that fewer THMs, HAA_5_, and total organic halogen (TOX) were generated in chloramination process (combined chlorine) than those in chlorination process (free chlorine) (Hua and Reckhow, 2008). The presence of [NH_4_^+^] also affected the ratio between THMs and HAA_5_. A high concentration of ammonia level (e.g., >5 mg/L as N) was reported to inhibit the production of THMs, while lower ammonia concentration (<0.5 mg/L as N) favored the THMs production in treated wastewater effluent (De Leer et al., 1990). Currently, there are no guidelines related to DPBs levels in wastewater reuse. In this study, the concentrations of THMs and HAA_5_ detected after EC disinfection were generally within the range of those reported in chorine disinfected secondary wastewater effluent (Bougeard et al., 2010, Krasner et al., 2009) or swimming pool waters (Lee et al., 2010). The result indicates the EC treated toilet wastewater should be safe for non-potable reuse applications from the aspect of DBPs.

## 5 Conclusions

• EC disinfection using WEC can effectively inactivate both viruses and bacteria in toilet wastewater without adding any supporting electrolytes. The system can be developed into a commercial viable self-sufficient solar-powered mobile toilet for decentralized wastewater treatment.
• Viruses were more resistant than bacteria in both EC and CC disinfection. A 5-log_10_ reduction of MS2 (the conservative model microorganism) in toilet wastewater can be achieved by EC disinfection at applied cell voltage of 4 V in 1 h, while CC disinfection is not effective for virus inactivation in toilet wastewater.
• RCS are the main disinfectants produced by the active bismuth-doped TiO_2_ anode. Ammonia can significantly reduce the disinfection efficiency by converting free chlorine to chloramines. The high inactivation rate of viruses with EC disinfection can be explained by the coexistence of free chlorine and free ammonia during EC reaction.
• Higher applied cell voltage and longer reaction time will generate more organic DBPs (THMs and HAA_5_). Most of the DBPs are formed during the EC reaction rather than the after treatment incubation period.
• The WEC treated toilet wastewater is safe for non-potable reuse, such as toilet flushing and agricultural irrigation.

## Figures and Tables

**Table 1:** Chemical parameters of toilet wastewater and model waters.

	pH	Electrical conductivity (mS/cm)	Cl^−^(mM)	NH_4_^+^(mM)
Toilet water	6.7–8.3	3.2–3.4	12–20	4.6–4.7
MW1	7.4–7.5	3.2–3.4	n.d.^a^	n.d.
MW2	7.4–7.5	3.2–3.4	15	n.d.
MW3	7.4–7.5	3.2–3.4	15	15

a n. d. – non-detectable.
