# Supplementary material for: Electrochemical disinfection of toilet wastewater using wastewater electrolysis cell
Source: Water Res. 2016 Apr 1;92:164–72. doi: 10.1016/j.watres.2016.01.040 (PMC4773403; doi:10.1016/j.watres.2016.01.040)
Supplement: Supplementary file 1 [file mmc1.docx]

**Supporting Information**

**Electrochemical Disinfection of Toilet Wastewater Using Wastewater Electrolysis Cell**

Xiao Huang^a^, Yan Qu^b^, Clément A. Cid^b^, Cody Finke^b^ , Michael R. Hoffmann^b^, Keahying Lim^a^, Sunny C. Jiang^a,*^

^a^ Civil and Environmental Engineering, University of California, Irvine, California 92697, United States

^b^ Linde+Robinson Laboratories, California Institute of Technology, Pasadena, California 91125, United States

*Corresponding author: Sunny C. Jiang

E-mail: sjiang@uci.edu

Address: 844 Engineering Tower

University of California, Irvine

The Henry Samueli School of Engineering

Irvine, CA 92697-7070

Tel: (949) 824-5527

Fax: (949) 824-3672

**Table S1**

Characteristics of raw toilet wastewater.

| Parameters | Value |
| --- | --- |
| pH | 6.7~8.3 |
| Conductivity (mS/cm) | 3.2~3.4 |
| DOC (mg/L) | 57.5 |
| Chloride (mM) | 12~20 |
| NH_4_^+^ (mM) | 4.5~4.7 |
| Total Culturable Bacteria (CFU/mL) | 3.2×10^5^ |
| Total Bacteria (Cells/mL by FCM counts) | 2.49×10^7^ |
| Total Viruses (Virus particles/mL by FCM counts) | 3.74×10^8^ |
| *E. coli* (CFU/mL using *mTEC*) | 4.1 |
| *Enterococcus* (CFU/mL using *m-EI*) | 6.4×10^2^ |


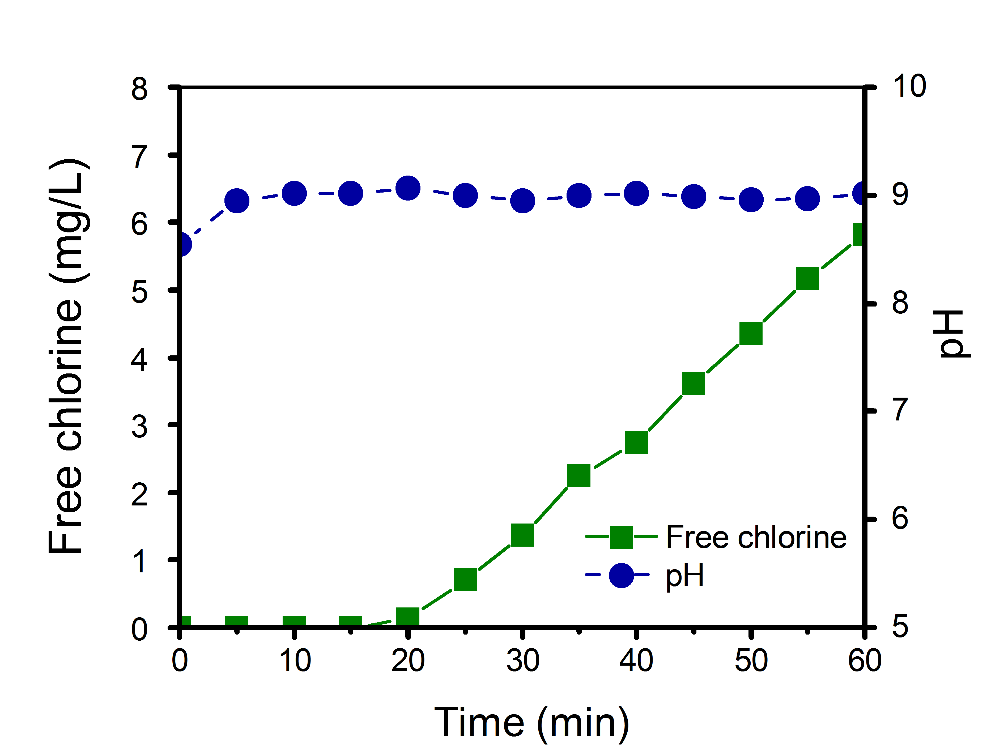


**Fig. S1** Free chlorine generation and pH changes in 20 L pilot scale wastewater electrolysis cell detected by online free chlorine and pH probes during toilet wastewater disinfection experiment (Applied cell voltage 3.8V).





**Fig. S2** Flow cytometry (FCM) density plots of florescence channel 1 *vs.* side scatter (FL1 *vs.* SSC) showing (a) *E. coli* and (b) *Enterococcus* before (left, 0min) and after (right, 30 min) electrochemical (EC) disinfection in toilet wastewater at applied cell voltage of 4V. Samples were stained with 0.5x SYBR-gold. Gate R1-R3 were used to differentiate different bacterial population based on their fluorescence intensity.
